# Effects of the New Aldose Reductase Inhibitor Benzofuroxane Derivative BF-5m on High Glucose Induced Prolongation of Cardiac QT Interval and Increase of Coronary Perfusion Pressure

**DOI:** 10.1155/2016/5281267

**Published:** 2015-12-29

**Authors:** C. Di Filippo, B. Ferraro, R. Maisto, M. C. Trotta, N. Di Carluccio, S. Sartini, C. La Motta, F. Ferraraccio, F. Rossi, M. D'Amico

**Affiliations:** ^1^Department of Experimental Medicine, Section of Pharmacology “L. Donatelli”, Second University of Naples, 80138 Naples, Italy; ^2^Department of Pharmacy, University of Pisa, 56126 Pisa, Italy; ^3^Department of Clinical, Public and Preventive Medicine, Second University of Naples, 80138 Naples, Italy

## Abstract

This study investigated the effects of the new aldose reductase inhibitor benzofuroxane derivative 5(6)-(benzo[*d*]thiazol-2-ylmethoxy)benzofuroxane (BF-5m) on the prolongation of cardiac QT interval and increase of coronary perfusion pressure (CPP) in isolated, high glucose (33.3 mM D-glucose) perfused rat hearts. BF-5m was dissolved in the Krebs solution at a final concentration of 0.01 *μ*M, 0.05 *μ*M, and 0.1 *μ*M. 33.3 mM D-glucose caused a prolongation of the QT interval and increase of CPP up to values of 190 ± 12 ms and 110 ± 8 mmHg with respect to the values of hearts perfused with standard Krebs solution (11.1 mM D-glucose). The QT prolongation was reduced by 10%, 32%, and 41%, respectively, for the concentration of BF-5m 0.01 *μ*M, 0.05 *μ*M, and 0.1 *μ*M. Similarly, the CPP was reduced by 20% for BF-5m 0.05 *μ*M and by 32% for BF-5m 0.1 *μ*M. BF-5m also increased the expression levels of sirtuin 1, MnSOD, eNOS, and FOXO-1, into the heart. The beneficial actions of BF-5m were partly abolished by the pretreatment of the rats with the inhibitor of the sirtuin 1 activity EX527 (10 mg/kg/day/7 days i.p.) prior to perfusion of the hearts with high glucose + BF-5m (0.1 *μ*M). Therefore, BF-5m supplies cardioprotection from the high glucose induced QT prolongation and increase of CPP.

## 1. Introduction

Hyperglycemia during the diabetes has detrimental effects on various organs including eye, kidney, central nervous system, and heart [1]. Hyperglycemia favors the insurgence of coronary artery disease, peripheral arterial diseases, cardiomyopathy, angina, and myocardial infarction [1]. Among these, the most dangerous consequence of the hyperglycemia is the prolongation of the cardiac QT interval, which leads diabetic patients to sudden death [2]. Long cardiac QT interval is partly sensitive to antioxidant drugs acting on the nitric oxide bioavailability, glycosylated products, accumulation of reactive oxygen species, and impairment of ionic pumps in models of isolated high glucose perfused heart [3, 4]. Increased interest, therefore, has gained the discovery of new drugs that may modulate pathways involved in glucose metabolism and hyperglycemia-induced modifications, in order to produce cardiovascular protection. In this context, one pathway that could be targeted is the aldose reductase (ALR2), because it contributes to the deleterious actions of hyperglycemia onto the cardiovascular system by inducing oxidative damage into the heart following diabetes [5–9].

Recently, Sartini et al. [10] discovered a series of new benzofuroxane derivatives which inhibits the aldose reductase (ALR2). These compounds spontaneously release NO, possess a hydroxyl radical scavenging activity, and account for multieffective agents for the treatment of cardiovascular diabetic complications. For this, the first aim of the study was to investigate the effects of the BF-5m, on the prolongation of cardiac QT interval and increase of the CPP induced by perfusion with high glucose of isolated rat heart.

In addition, since the action of ALR2 on glucose metabolism is linked to depletion into the cells of the cofactor NAD+ [11] and SIRT1 is a NAD1-dependent protein deacetylase which belongs to a class of proteins that lead to improved energy consumption, limitation of oxidative stress, and reduced DNA damage [12, 13], the second aim of the study was to investigate whether there is an involvement of the sirtuin 1 (SIRT1) in the cardiac effects of BF-5m. SIRT1 is one of the better characterized sirtuins with multiple protective actions in many pathological conditions, through involvement of several molecular pathways, deacetylation of mediators of oxidative stress, inflammation, apoptosis, and transcription factors, and plays an important role in the regulation of glucose consumption by regulating insulin expression in vivo [14, 15]. SIRT1 also has strong antioxidant action [16].

## 2. Material and Methods

### 2.1. Drug

BF-5m, 5(6)-(benzo[*d*]thiazol-2-ylmethoxy)benzofuroxane, was synthesized at the Department of Pharmacy of the University of Pisa, Italy, following a previously reported procedure [10]. Briefly, alkylation of the commercially available 4-amino-3-nitrophenol with chloroacetonitrile, in the presence of anhydrous potassium carbonate, provided the 2-(4-amino-3-nitrophenoxy)acetonitrile which, through reaction with o-aminothiophenol, gave the key intermediate 4-[(benzo[*d*]thiazol-2-yl)methoxy]-2-nitrobenzenamine. Treatment with sodium nitrite in concentrated hydrochloric acid and then with sodium azide in water gave the corresponding azido-derivative, which cyclized to the target inhibitor, 5(6)-(benzo[*d*]thiazol-2-ylmethoxy)benzofuroxane, when heated under reflux in acetic acid.

### 2.2. Isolated Heart Preparation

Male Sprague-Dawley rats (3-4 months old, with a weight of 210 ± 20 g) were anaesthetised with urethane (1.2 mg/kg i.p.) and heparinized (sodium heparin, 250 IU, i.p. 10 min before heart removal). Subsequently, the hearts were quickly excised and placed in ice-cold Krebs solution (composition in mmol/L: 11.1 D(+)-glucose; 1.4 CaCl_2_; 118.5 NaCl; 25.0 NaHCO_3_; 1.2 MgSO_4_; 1.2 NaH_2_PO_4_; 4.0 KCl). Then, the hearts were connected to a Langendorff apparatus via the aorta and retrogradely perfused at 37°C under constant flow (9-10 mL/min) with the Krebs solution bubbled with 95% O_2_-5% CO_2_ and composed as described above.

A total of 82 hearts were used. Of these, 12 were excluded having a sinus rate of <210 beats per minute or a coronary perfusion pressure (CPP) <60 mmHg between 5 and 15 min after beginning of the perfusion; hearts not in sinus rhythm during the study were also excluded. The remaining 70 hearts having a sinus rate of >210 beats per minute or CPP > 60 mmHg, between 5 and 15 min after beginning, were used in the study and divided into the following experimental groups (*n* = 10 rats for each group): (i) control: hearts perfused for 2 hours with a Krebs solution containing D-glucose at 11.1 mM [4, 5]; (ii) high glu: hearts perfused for 2 hours with Krebs solution containing D-glucose at 33.3 mM [4, 5]; (iii) high glu + DMSO: hearts perfused with Krebs solution containing 1% DMSO + D-glucose at 33.3 mM; (iv) high glu + BF-5m: hearts perfused for 2 hours with Krebs solution containing D-glucose at 33.3 mM + BF-5m (0.01 *μ*M in 1% DMSO); (v) high glu + BF-5m: hearts perfused for 2 hours with Krebs solution containing D-glucose at 33.3 mM + BF-5m (0.05 *μ*M, in 1% DMSO); (vi) high glu + BF-5m: hearts perfused for two hours with Krebs solution containing D-glucose at 33.3 mM + BF-5m (0.1 *μ*M, in 1% DMSO). Moreover, in order to assess the role of SIRT1 in the BF-5m cardiac effects additional studies were done on 10 hearts excised from rats pretreated for 7 days (10 mg/kg/day i.p.) [17] with EX527 and perfused with a Krebs solution with high glucose + BF-5m 0.1 *μ*M. In these the QT interval, CPP values, and biochemical parameters mentioned below were monitored.

### 2.3. QT Interval and CPP Measurements

QT interval, for each heart, was recorded by a unipolar ECG with a stainless steel wire electrode in the apex of left ventricular muscle mass, with a second electrode connected to the aorta. This electrode arrangement gave clear P waves and ventricular complexes. An ECG (chart speed 50 mm/s) was recorded for 4 min every 10 min for 2 h. The ECG was analysed and heart rate (RR interval) and the width of the ventricular complex (QT) at 100% repolarization (QT100) were measured. The CPP in the aortic line was monitored using a Statham Spectramed pressure transducer connected to a chart recorder (Grass, 79E, Quincy, MA, USA). Temperature was maintained constant by means of a heated (37°C) water jacket. On establishing a stable value (20–30 min following cannulation) CPP was calculated according to Di Filippo et al. [4]. Briefly, CPP value was expressed either as the mean of each 10-min value throughout the entire experiment or as the mean of the steady-state increment above baseline when an increase of CPP was evident during an experiment. All experimental procedures were approved by Animal Care Ethical Committee of the Naples University in accordance with Italian (Decree 116/92) and European Community (E.C. L358/1 18/12/86) guidelines on the use and protection of laboratory animals.

### 2.4. Hematoxylin and Eosin Staining

At the end of the perfusion period the hearts were cut into two halves and one was immediately frozen in liquid nitrogen and stored at −80°C. The hematoxylin and eosin staining was conducted according to Marfella et al. [18] protocol and 200x magnification pictures were taken.

### 2.5. Western Blot Analysis and SIRT1 Activity Assay

Western blotting analysis was performed from the homogenized frozen biopsy according to Di Filippo et al. [19]. We used (i) primary anti-SIRT1 antibody (1 : 1000, Abcam, Cambridge, UK), (ii) specific monoclonal antibody directed against FOXO-1 (1 : 500, Millipore, California, USA), (iii) anti-MnSOD primary antibody (1 : 800, Millipore, California, USA), (iv) anti-*β*-actin monoclonal antibody (1 : 1000, Sigma-Aldrich), and (v) anti-eNOS sc-654 (1 : 500, Santa Cruz Biotech, USA) with an enhanced chemiluminescence detection reagent (ECL), quantified by densitometry using a BioRad ChemiDoc MP Imaging system. The following secondary antibodies were used: goat anti-mouse (1 : 1000, Santa Cruz Biotech, USA) and goat anti-rabbit (1 : 1000, Santa Cruz Biotech, USA). The deacetylase activity of SIRT1 was measured by means of a commercial fluorometric kit (Abcam, Cambridge, UK) and normalized by protein content.

### 2.6. Sorbitol Assay in Heart Samples

A procedure early described in literature [20] with slight modifications was used to assess the sorbitol levels in the heart homogenates by fluorimetry. Briefly, one mL of the supernatants was incubated for 5 min at 37°C in presence of 50 *μ*mol glycine buffer, 2 *μ*mol magnesium chloride, and 0.2 *μ*mol nicotinamide adenine dinucleotide (NAD) and reaction initiated by the addition of 0.6 U of sorbitol dehydrogenase. A standard curve was constructed with sorbitol from 0.4 to 10 *μ*g/mL; the fluorescence from NADH formation was measured at 450 nm and expressed as sorbitol *μ*g/mL.

### 2.7. RNA Isolation and Quantification

Total RNA was extracted from the heart tissue (~50 mg) using the Rneasy Plus Mini Kit (Qiagen) according the manufacturer's protocol from Animal Cells. Then RNA was quantified using NanoDrop 2000c Spectrophotometer (Thermo Scientific, Waltham, MA, USA) according to Rossi et al. [21].

### 2.8. RNA Retrotranscription and Real Time PCR Reaction

cDNA synthesis was obtained using SuperScript III Reverse Transcriptase Kit (Invitrogen, USA) starting from 1.5 *μ*g of total RNA. In the subsequent q-PCR reaction performed with Power SYBR Green PCR Master Mix (Applied Biosystems, UK), SIRT1 mRNA has been quantified and normalized using *β*-actin as housekeeping gene in CFX96 Real Time System (BioRad) cycler (BioRad Laboratories, Inc.). Primers sequences were as follows: 5′-TGTTTCCTGTGGGATACCTGA-3′ (sense) and 5′-TGAAGAATGGTCTTGGGTCTTT-3′ (antisense) for SIRT1 and 5′-CGAGTACAACCTTCTTGCAG-3′ (sense) and 5′-TTCTGACCCATACCCACCAT-3′ (antisense) for *β*-actin. Relative quantification of gene expression was normalized to beta-actin using the 2^−ΔΔCt^ method [19, 21].

### 2.9. Statistical Analysis

Data are expressed as means ± standard error of the mean (s.e.m.). Student's *t*-test (when only two groups were compared) or one-way ANOVA followed by Dunnett's test (more than two experimental groups) was used. *P* < 0.05 was considered statistically significant.

## 3. Results

**Figure 1:**
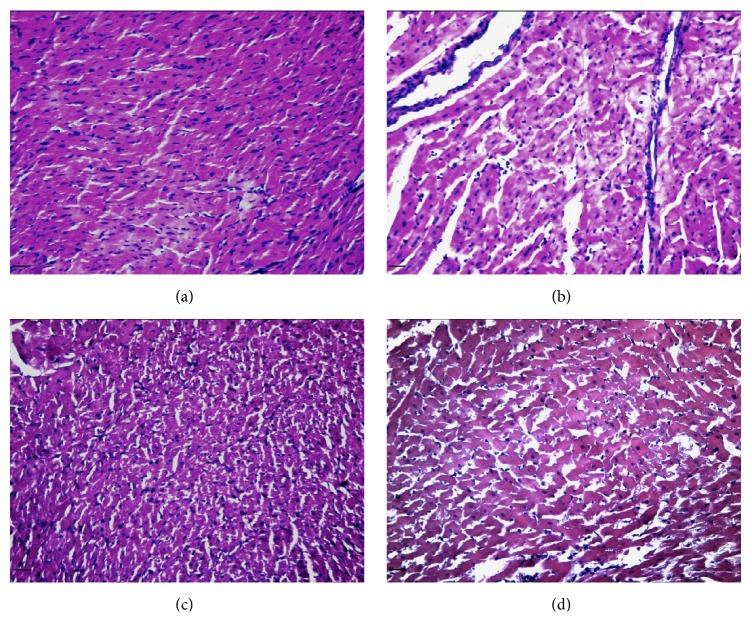
Representative pictures from hematoxylin and eosin staining of cardiac tissue. Rat hearts were perfused with Krebs solution containing (a) D-glucose 11.1 mM (control); (b) glucose 33.3 mM (high glu); (c) high glu + BF-5m (0.1 *μ*M); (d) high glu + BF-5m (0.1 *μ*M) + EX527 (10 mg/kg/day i.p.). 200x magnification; scale bar = 100 *μ*m.

Figure 1 shows representative pictures of the heart structure derangement caused by perfusion of the hearts with the a Krebs solution containing high glucose concentration (D-glucose, 33.3 mM) with respect to the hearts perfused with a Krebs solution containing normal glucose concentration (D-glucose, 11.1 M), in presence or absence of the first active dose of the compound BF-5m (e.g., 0.05 *μ*M). There was clear evidence of a damaged structure with no or few signs of well tissue organization (e.g., bands, Z line) following high glucose perfusion. In contrast, a discrete preservation of the heart structure was seen in rat hearts perfused with high glucose + BF-5m with an almost intact structure of the tissue. This cardiac preservation disappeared when the rats were pretreated with the SIRT1 activity inhibitor EX527 (10 mg/kg/day/7 days i.p.) and then perfused with high glucose + BF-5m 0.1 *μ*M (Figure 1).

### 3.1. BF-5m Decreased QT Interval and CPP in Rats Hearts Perfused with the High Glucose Concentration

**Figure 2:**
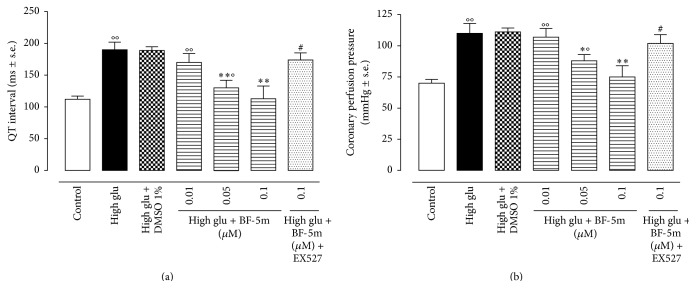
(a) Cardiac QT interval in hearts of Sprague-Dawley rats perfused for two hours in a Langendorff apparatus with Krebs solution containing D-glucose 11.1 mM (control); glucose 11.1 mM + DMSO 1% (vehicle); glucose 33.3 mM (high glu); high glu + BF-5m (0.01, 0.05, and 0.1 *μ*M); high glu + BF-5m (0.1 *μ*M) + EX527 (10 mg/kg/day i.p.). (b) Coronary perfusion pressure (CPP) recorded on a Power Lab system following exposure of perfused hearts to the same treatments as in panel (a). Values are expressed as the mean of 10 observations ± s.e.m. Significant differences* versus* control are reported as °*P* < 0.05 and °°*P* < 0.01; significant differences* versus* high glu are reported as ^*∗*^ *P* < 0.05 and ^*∗∗*^ *P* < 0.01; significant differences* versus* high glu + BF-5m 0.1 *μ*M are reported as ^#^ *P* < 0.01.

11.1 mM glucose (control) exhibited a QT interval of 112 ± 5 ms and a CPP of 70 ± 3 mmHg. These values were increased up to 190 ± 12 ms (*P* < 0.01 versus control) for QT and up to 110 ± 8 mmHg (*P* < 0.01 versus control) for CPP in the heart perfused with glucose 33.3 mM. The addition of BF-5m (0.01 *μ*M; 0.05 *μ*M; 0.1 *μ*M) to Krebs solution containing a high glucose concentration diminished the QT interval of 10%, 32%, and 41% with calculated values of 170 ± 14 ms, 130 ± 12 ms, and 113 ± 20 ms, respectively. Similarly, the CPP was reduced by 3% (107 ± 12 mmHg), 20% (88 ± 5 mmHg), and 32% by BF-5m (Figure 2). Pretreatment (7 days) of the rats with EX527 prior to the perfusion of the hearts with high glu + BF-5m 0.1 *μ*M decreased the BF-5m cardioprotection by reporting the QT interval at 174 ± 11 ms (*P* < 0.01) and CCP at 102 ± 7 mmHg (*P* < 0.01 versus high glu + BF-5m 0.1 *μ*M) (Figure 2).

### 3.2. Effects of BF-5m on SIRT1 Levels and Activity in Rats Hearts Perfused with the High Glucose Concentration

**Figure 3:**
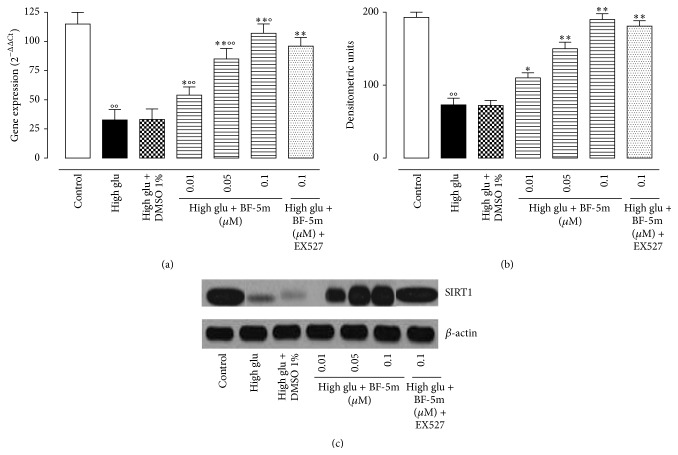
(a) SIRT1 in perfused hearts following treatment with D-glucose 11.1 mM (control); glucose 11.1 mM + DMSO 1% (vehicle); glucose 33.3 mM (high glu); high glu + BF-5m (0.01, 0.05, and 0.1 *μ*M); high glu + BF-5m (0.1 *μ*M) + EX527 (10 mg/kg/day i.p.). Total RNA was extracted from the hearts and reverse transcribed into cDNA using superscript reverse transcriptase system. The expression of SIRT1 was quantified by qPCR using commercially available rat primers. Results are expressed as arbitrary units based on calculation of 2^−ΔΔCt^ method. °*P* < 0.01* versus* control; ^*∗*^ *P* < 0.05 and ^*∗∗*^ *P* < 0.01* versus *high glu. (b) Addition of BF-5m (0.01, 0.05, and 0.1 *μ*M) to the high glucose Krebs solution dose-dependently increased the expression of SIRT1. Results are derived from western blotting (panel (c) representative) and expressed as densitometric units (mean ± s.e.m. of *n* = 10 observations for each group). Significant differences* versus* control are reported as °*P* < 0.05 and °°*P* < 0.01; significant differences* versus* high glu are reported as ^*∗*^ *P* < 0.05 and ^*∗∗*^ *P* < 0.01; significant differences* versus* high glu + BF-5m 0.1 *μ*M are reported as °*P* < 0.01.

Figure 3 showed that SIRT1 gene and protein expression significantly decreased in rat hearts perfused for two hours with Krebs solution containing a high glucose concentration (*P* < 0.01 versus control). Addition of BF-5m at 0.01, 0.05, and 0.1 *μ*M to the Krebs solution plus glucose at 33.3 mM significantly increased the cardiac SIRT1 gene expression (e.g., +65%, +160%, and +227%) and protein levels (e.g., +50%, +105%, and +160%; Figure 3). SIRT1 deacetylase activity was significantly increased in heart homogenates from high glu + BF-5m with respect to the activity measured in hearts from high glu. The SIRT1 deacetylase activity was 450 ± 32 RFU/*μ*g of protein in high glu perfused hearts; 492 ± 48 RFU/*μ*g of protein in high glu + BF-5m 0.01 *μ*M; 726 ± 64 RFU/*μ*g of protein in high glu + BF-5m 0.51 *μ*M; 1149 ± 86 RFU/*μ*g of protein in high glu + BF-5m 0.1 *μ*M.

EX527 pretreatment (10 mg/kg/day/7 days i.p.) did not affect SIRT1 gene and protein expression levels but diminished (−57%) the BF-5m cardioprotection (Figures 1 and 2).

### 3.3. Effects of BF-5m on MnSOD, eNOS Expression, and Tissue Sorbitol Content

**Figure 4:**
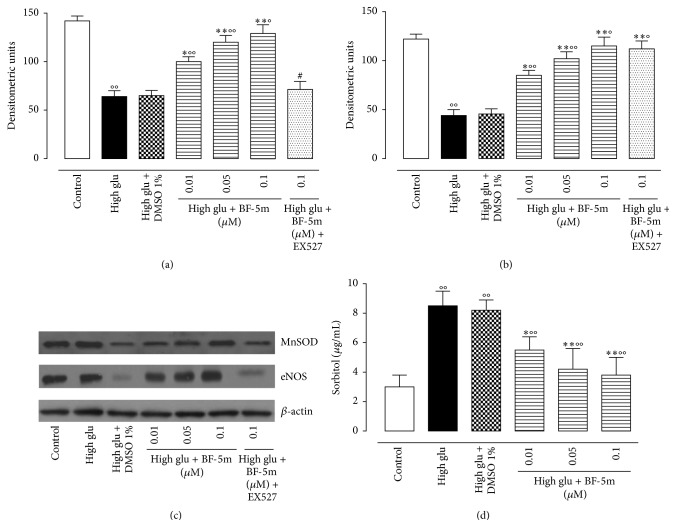
(a) MnSOD expression in hearts perfused with glucose 11.1 mM (control); glucose 11.1 mM + DMSO 1% (vehicle); glucose 33.3 mM (high glu); high glu + BF-5m (0.01, 0.05, and 0.1 *μ*M); high glu + BF-5m (0.1 *μ*M) + EX527 (10 mg/kg/day i.p.). (b) eNOS expression levels in hearts perfused as in panel (a). Results are derived from western blotting (panel (c) representative) and expressed as densitometric units; (d) tissue sorbitol content (*μ*g/mL). Mean ± s.e.m. of *n* = 10 observations for each group. Significant differences* versus* control are reported as °*P* < 0.05 and °°*P* < 0.01; significant differences* versus* high glu are reported as ^*∗*^ *P* < 0.05 and ^*∗∗*^ *P* < 0.01; significant differences* versus* high glu + BF-5m 0.1 *μ*M are reported as ^#^ *P* < 0.01.

As shown in Figures 4(a)–4(c), the perfusion of the hearts with high glu + BF-5m (0.01 *μ*M; 0.05 *μ*M; 0.1 *μ*M) increased the levels of MnSOD and eNOS compared to the hearts perfused with the high glucose only. In contrast, the effects of 0.1 *μ*M BF-5m on MnSOD and eNOS were decreased by the EX527 pretreatment (Figures 4(a)–4(c)). The tissue sorbitol content as marker of the aldose reductase activity was increased in heart perfused with high glu with respect to the content of vehicle. This increase was not observed after the treatment of the rats with the BF-5m at all the concentration used (Figure 4(d)).

### 3.4. Effect of BF-5m on FOXO-1

**Figure 5:**
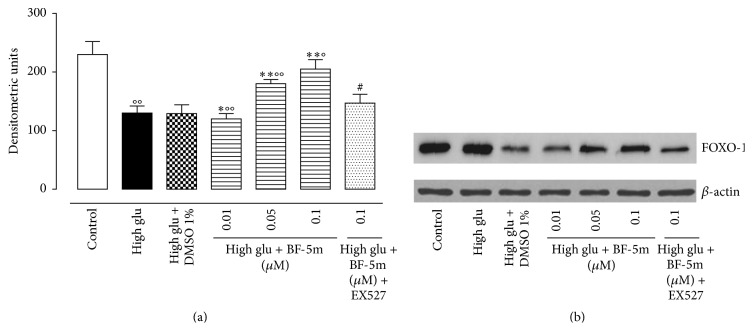
(a) Expression of FOXO-1 in hearts perfused with glucose 11.1 mM (control); glucose 11.1 mM + DMSO 1% (vehicle); glucose 33.3 mM (high glu); high glu + BF-5m (0.01, 0.05, and 0.1 *μ*M); high glu + BF-5m (0.1 *μ*M) + EX527 (10 mg/kg/day i.p.). Results are derived from western blotting (panel (b) representative) and expressed as densitometric units and represented the mean ± s.e.m. of *n* = 10 observations for each group. Significant differences* versus* control are reported as °*P* < 0.05 and °°*P* < 0.01; significant differences* versus* high glu are reported as ^*∗*^ *P* < 0.05 and ^*∗∗*^ *P* < 0.01; significant differences* versus* high glu + BF-5m 0.1 *μ*M are reported as ^#^ *P* < 0.01.

BF-5m modified the levels of cardiac FOXO-1 (Forkhead transcription factor 1), which is a direct target of SIRT1. Western blotting analysis showed lower expression of this protein in hearts perfused with high glucose solution. This was reported towards the control values by high glu + BF-5m (Figure 5). The inhibitor of SIRT1 activity EX527 also inhibited the restoring of FOXO-1 levels operated by the BF-5m (Figure 5).

## 4. Discussion

Here we show that inhibition of the endogenous enzyme aldose reductase (ALR2) activity by the newly synthetized benzofuroxane derivative 5(6)-(benzo[*d*]thiazol-2-ylmethoxy)benzofuroxane (BF-5m) results in cardioprotection from the electrical instability and increased vasomotor tone caused by high levels of glucose into the heart. This cardioprotection is characterized by reduction of the long cardiac QT interval and the decrease of the coronary perfusion pressure (CPP). The ALR2 is a critical enzyme when there is a high glucose condition into cells and tissues since this by catalyzing the reduction of glucose to sorbitol [22] favors accumulation of this polyol into the cell cytoplasm of organs and tissues and determines local generation of reactive oxygen species and damage [22]. Over the years many compounds have shown potent inhibitory effects against the enzyme aldose reductase (ALR2) including, for example, epalrestat, fidarestat, lidorestat, and sorbinil [22–24]. However, some of these were withdrawn from clinical trials because they showed undesirable effects such as skin reactions or liver toxicity [25]. Numerous efforts have been made, therefore, to identify molecules that could effectively block the activity of ALR2, limit the negative effects from prolonged exposure to high glucose, and possibly have few or no side effects. Among these, Sartini et al. [10] proposed a novel class of nonhydantoin noncarboxylic acid inhibitors, featuring the benzofuroxane core [22, 26] as new scaffold interacting with the so-called ALR2 anion site. Merging submicromolar ALR2 inhibitory activities with significant ROS scavenging properties, these compounds have been identified as the ideal therapeutic treatment for the high glucose-related pathologies [10] as could be the alterations of cardiac electrical stability. Effectively, BF-5m reduced the prolongation of cardiac QT interval, sensitive marker of electrical instability, in our setting.

BF-5m also promotes increase of the expression and activity of endogenous antioxidant pathways and free radical scavengers such as SIRT1 and MnSOD, its downstream target [27], into the heart following exposure to a high glucose stimulus. Indeed, the high glucose to the heart caused decrease of the protein SIRT1 into the tissue, an effect that was reverted by the BF-5m. SIRT1 is NAD1-dependent protein deacetylase which belongs to a class of proteins that lead to improved energy consumption, limitation of oxidative stress, and reduced DNA damage [12, 13]. SIRT1, through involvement of several molecular pathways and deacetylation of mediators of oxidative stress, inflammation, apoptosis, and transcription factors, possesses multiple protective actions in many pathological conditions. SIRT1 also plays an important role in the regulation of insulin expression by glucose concentration [14, 15]. So, back to our game, BF-5m was able to reduce the structural and functional cardiac derangement caused by high glucose into the myocardium through the modulation of SIRT1 activity. This result is also confirmed by the reduction in the expression of the Forkhead transcription factor 1 (FOXO-1), the direct downstream product of SIRT1 activity into tissues. FOXO-1 is a transcription factor that is regulated by SIRT1 through its deacetylase activity [28, 29] and it regulates gluconeogenesis, glycogenolysis, and adipogenesis [30]. From the clinical point of view, upregulated FOXO-1 raises a state of insulin resistance and diabetes, due to a loss of insulin sensitivity, and the consequent hyperglycemia or high glucose accelerates cellular damage [31]. In our case, a condition of diminished activity of FOXO-1 caused by high glucose may be one of the actors accounting for an altered homeostasis at cardiac levels which may have led to reduced perfusion of heart and finally electrical instability.

## 5. Conclusions

These results suggest that the new aldose reductase inhibitor benzofuroxane derivative BF-5m supplies cardioprotection from the high glucose induced QT prolongation and increase of CPP. The mechanism of action involves the increase of SIRT1 protein and activity into the heart tissue, together with the modulation of the expression and activity of its downstream mediators MnSOD and FOXO-1.
